# Phosphatidyl Inositol 3-Kinase (PI3K)-Inhibitor CDZ173 protects against LPS-induced osteolysis

**DOI:** 10.3389/fphar.2022.1021714

**Published:** 2023-01-06

**Authors:** Zuoxing Wu, Xuedong Li, Xiaohui Chen, Xuemei He, Yu Chen, Long Zhang, Zan Li, Mengyu Yang, Guixin Yuan, Baohong Shi, Ning Chen, Na Li, Haotian Feng, Mengyu Zhou, Gang Rui, Feng Xu, Ren Xu

**Affiliations:** ^1^ Department of Orthopedic Surgery, The First Affiliated Hospital of Xiamen University, School of Medicine, Xiamen University, Xiamen, China; ^2^ The First Affiliated Hospital of Xiamen University-ICMRS Collaborating Center for Skeletal Stem Cell, School of Medicine, Xiamen University, Xiamen, China; ^3^ Fujian Provincial Key Laboratory of Organ and Tissue Regeneration, School of Medicine, Xiamen University, Xiamen, China; ^4^ Department of Medical Laboratory, The Fourth Affiliated Hospital of Guangxi Medical University, Liuzhou, China; ^5^ Department of Sports Medicine, Xiangya Hospital, Central South University, Changsha, China; ^6^ Department of Endocrinology, Zhongshan Hospital (Xiamen), Fudan University, Xiamen, China; ^7^ Inner Mongolia Dairy Technology Research Institute Co. Ltd., Hohhot, China; ^8^ Department of Dentistry, The First Affiliated Hospital of Guangxi Medical University, Nanning, China; ^9^ Department of Subject Planning, Ninth People’s Hospital Shanghai, Jiaotong University School of Medicine, Shanghai, China; ^10^ Research Centre for Regenerative Medicine, Guangxi Key Laboratory of Regenerative Medicine, Guangxi Medical University, Nanning, China

**Keywords:** Osteoclast, CDZ173, Osteolysis, PI3K, MAPK

## Abstract

A major complication of a joint replacement is prosthesis loosening caused by inflammatory osteolysis, leading to the revision of the operation. This is due to the abnormal activation of osteoclast differentiation and function caused by periprosthetic infection. Therefore, targeting abnormally activated osteoclasts is still effective for treating osteolytic inflammatory diseases. CDZ173 is a selective PI3K inhibitor widely used in autoimmune-related diseases and inflammatory diseases and is currently under clinical development. However, the role and mechanism of CDZ173 in osteoclast-related bone metabolism remain unclear. The possibility for treating aseptic prosthesis loosening brought on by inflammatory osteolysis illness can be assessed using an LPS-induced mouse cranial calcium osteolysis model. In this study, we report for the first time that CDZ173 has a protective effect on LPS-induced osteolysis. The data show that this protective effect is due to CDZ173 inhibiting the activation of osteoclasts *in vivo*. Meanwhile, our result demonstrated that CDZ173 had a significant inhibitory effect on RANKL-induced osteoclasts. Furthermore, using the hydroxyapatite resorption pit assay and podosol actin belt staining, respectively, the inhibitory impact of CDZ173 on bone resorption and osteoclast fusion of pre-OC was determined. In addition, staining with alkaline phosphatase (ALP) and alizarin red (AR) revealed that CDZ173 had no effect on osteoblast development *in vitro*. Lastly, CDZ173 inhibited the differentiation and function of osteoclasts by weakening the signal axis of PI3K-AKT/MAPK-NFATc1 in osteoclasts. In conclusion, our results highlight the potential pharmacological role of CDZ173 in preventing osteoclast-mediated inflammatory osteolysis and its potential clinical application.

## Introduction

Osteoclasts and osteoblasts work together to maintain the integrity and health of bone tissue through a dynamic equilibrium in the metabolism of bone tissue (Rossi et al., 2019; Zhan et al., 2019). Osteolysis is a pathological condition characterized by bone loss and osteopenia caused by excessive activation of osteoclasts or reduction of osteoblasts. The diseases associated with it include osteoporosis, aseptic prosthesis loosening, periodontitis, and so on (Mbalaviele et al., 2017; Gao et al., 2022). Osteoclasts are the only multinucleated macrophages with bone resorption function in the body, which play a key role in bone development, reconstruction, and repair (Mediero and Cronstein, 2013; Yu et al., 2021). Joint replacement been extensively used to treat joint pain and joint instability caused by severe trauma, osteoarthritis and osteoporotic fractures (Wu et al., 2019; Zhou et al., 2020). However, aseptic loosening and periprosthetic infection leading to inflammatory osteolysis are still common complications of joint replacement, resulting in a high incidence rate and a decline in functional prognosis (Ren et al., 2011; Zhu et al., 2016). A primary reason for these inflammatory responses is that the excessive activation of osteoclasts induced by bacterial endotoxin pollution or bacterial endotoxin on implant-derived wear particles eventually leads to the loss of bone around the prosthesis (Han et al., 2020). Therefore, drug development targeting osteoclasts remains an effective means to prevent periprosthetic osteolysis.

From the hematopoietic stem cell lineage, osteoclasts are differentiated from mononuclear macrophages. Receptor activator of nuclear factor-κB ligand (RANKL) and macrophage colony-stimulating factor (M-CSF) play two important roles in osteoclast differentiation (Kim et al., 2020; Honma et al., 2021; Udagawa et al., 2021). As a member of the tumor necrosis factor superfamily, RANKL is a type II homotrimeric transmembrane protein that is mainly produced by osteoblasts and stromal cells in the form of surface proteins or secreted factors. RANK is a protein receptor highly expressed on the surface of osteoclasts. In the process of osteoclast maturation, the combination of RANKL and RANK will recruit some factors such as molecules like necrosis factor receptor-associated factor 6 (TRAF6) and Src tyrosine kinase. This recruitment will then activate downstream PI3K-AKT, NF-κB, and MAPK(An et al., 2019; Xin et al., 2020; Ma et al., 2021), thereby inducing the activation of the nuclear factor of activated T-cell cytoplasmic 1 (NFATc1), resulting in the subsequent expression of osteoclast-specific genes *DC-STAMP*, *MMP9*, *TRAP and CTSK* (Yang et al., 2019; Xin et al., 2020).

Lipopolysaccharide (LPS), a crucial part of Gram-negative bacteria’s cell membrane and a key biological agent thought to be responsible for inflammatory bone loss (Xing et al., 2011). Importantly, triggering an inflammatory response cascade can lead to the development of osteoclasts and an increase in bone loss. As a result, LPS can stimulate osteoclasts to activate NF-kB and MAPK signaling pathways by attaching to the pattern recognition receptor toll-like receptor (TLR4) (Shao et al., 2019; Fang et al., 2021). Simultaenously, LPS can induce systemic inflammatory responses and promote the production of osteoclast-related factors, such as TNF-α, MCSF, *etc.*, ultimately promoting the excessive activation of osteoclasts and bone loss (Wang et al., 2019). Therefore, developing drugs capable of inhibiting LPS-induced osteolysis remains a significant goal in preventing periprosthetic infection and inflammatory local bone destruction in aseptic joint loosening.

CDZ173 is a highly selective inhibitor of PI3K(Hoegenauer et al., 2017). In previous clinical studies, oral CDZ173 can effectively reduce lymphocyte proliferation dose-dependently to improve immune-related diseases (Rao et al., 2017). Systemic lupus erythematosus and rheumatoid arthritis are two examples of autoimmune disorders that can be made worse by activating the PI3K pathway. The development of CDZ173, as a potent and specific inhibitor of PI3K, has also been focused on investigating its benefits in the above diseases (Hoegenauer et al., 2017; Rao et al., 2017; Jamee et al., 2020) Meanwhile, studies have shown that CDZ173 can effectively inhibit the production of antigen-specific antibodies and alleviate disease symptoms in a rat collagen-induced arthritis model (Hoegenauer et al., 2017). However, the role of CDZ173 in osteolytic inflammatory diseases and osteoclasts has not been elucidated. Given the extensive research on CDZ173 in various immune diseases and its therapeutic potential in clinical trials, we explored the effect of CDZ173 on LPS-induced inflammatory bone loss and analyzed its specific cellular and molecular mechanisms.

## Materials and methods

### Reagents

**FIGURE 1:**
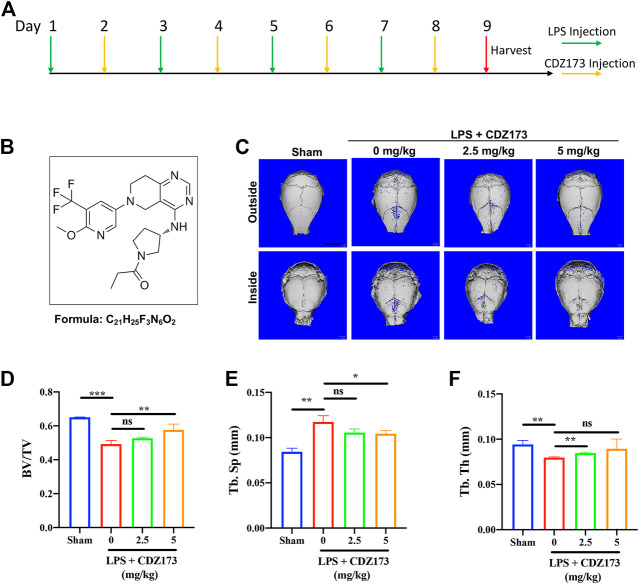
CDZ173 protected against titanium LPS-induced osteolysis of the mouse calvaria. **(A)** The timeline of the animal model. **(B)** Chemical structure of CDZ173. **(C)** Three-dimensional reconstructed micro-CT images of calvarial tissue from the Sham, PBS vehicle, and CDZ173 treated groups. **(D)** Bone volume to tissue volume (BV/TV,%) histomorphometric analysis **(E)** bone trabecular thickness **(F)** number of treatment group bone porosity (scale bar = 5 mm) (*p < 0.05, **p < 0.01, ***p < 0.001).

CDZ173 (purity >98% Figure 1A) was purchased from Selleck Co., LTD. (Shanghai, China). Recombinant M-CSF and RANKL mice were obtained from R&D Systems (Minneapolis, MN, United States). LPS was purchased from Sigma Chemical (Co.St.Louis, MO, United States). Promega (Madison, WI, United States) provided cell counting kits (CCK8). Tartrate-resistant acid phosphatase (TRAP) staining kits were obtained from Sigma Aldrich (St Louis, MO, United States). Antibodies for PI3K, phospho-PI3K, AKT, phospho-AKT, ERK, phospho-ERK, JNK, phospho-JNK, p38, phospho-p38, NF-κB p65, anti-phospho-NF-κB p65, β-Actin were obtained from Cell Signalling Technology (Boston, MA, United States). Antibodies for NFATc1 were obtained from Santa Cruz Biotechnology (Dallas, TX, United States), and antibodies for c-Fos were obtained from Abcam Technology (Cambridge, United Kingdom).

### Mice model

We randomly divided 32 healthy male C57BL/6J mice (6–8 weeks old) into 4 groups, namely the Sham group, LPS group, Low-Dose (2.5 mg/kg) group, and High-Dose (5 mg/kg) group, resulting in 8 mice in each group. After the start of the experiment, LPS was injected subcutaneously *via* the sagittal line of the skull in LPS group, low-dose (2.5 mg/kg) and high-dose (5 mg/kg) mice on the first, third, fifth and seventh day respectively, with the dose of 5 mg/kg LPS; On the second, fourth, sixth and eighth days, the mice in the low dose group (2.5 mg/kg) and the high dose group (5 mg/kg) were injected 2.5 mg/kg and 5 mg/kg CDZ173 through the sagittal line skin of the skull, respectively. Mice in the Sham group and LPS group were injected with the same amount of normal saline at the same position. Mice were slaughtered after the experiment on the ninth day, and their heads were removed before being preserved in 4% paraformaldehyde for 48 h and analyzed using microcomputed tomography (CT) and tissue sectioning.

### Micro-CT scanning and histological

The mouse skull specimens fixed with 4% FPA were scanned by high-resolution μCT. The set parameters were: voltage 50 kV and current 500 kV μ A. Scanning range: 2 cm × 2 cm, scanning layer thickness 10 μm. After scanning (Skyscan 1,272; Skyscan; Aartselaar, Belgium), three-dimensional reconstruction was performed with the nrecon software, and ctan software was used to analyze and compare the relevant parameters of reconstructed bone tissue, including bone volume fraction (BV/TV), trabecular bone separation (Tb. Sp), and trabecular bone porosity. The cranial bone samples after uCT scanning were placed in 10% EDTA and decalcified at room temperature for 4 weeks. 5 um sections were taken for Hematoxylin-Eosin (H&E) and Tartrate-resistant acid phosphatase (TRAP) activity staining. Hematoxylin-eosin staining is one of the most commonly used staining methods in paraffin section technology. The hematoxylin staining solution was alkaline, which mainly stained the chromatin in the nucleus and the nucleic acid in the cytoplasm with violet blue. Eosin is an acidic dye that mainly makes the cytoplasm and extracellular matrix components red. We observed the degree of bone destruction by H&E staining. Tartrate-resistant acid phosphatase (TRAP) staining here mainly explores the relative abundance of osteoclasts in bone tissue. Osteomeasure (Osteomeasure, LA, United States) was used to determine the number of osteoclast positive cells (TRAP + Cell Number) and the ratio of osteoclast to bone tissue contact surface (OC.S/BS) in each sample.

### Cell culture and CCK8 cell proliferation assay

In the first step, we obtained bone marrow monocyte/macrophages (BMMs) from long bone in 6–8-week-old male C57BL/6 mice. BMMs were grown in a humidified incubator at 37°C and 5% CO_2_ using a complete medium consisting of a-MEM supplemented with 10% (v/v) FBS, 1% (w/v) penicillin/streptomycin, and 30 ng/ml M-CSF. The CCK8 assay evaluated the effect of CDZ173 on the proliferation of BMMs cells. BMMs were seeded into a 96-well plate at a density of 6×10^3^ cells/well in full -MEM with 30 ng/mL M-CSF for 24 h. After that, the cells were incubated with the stated doses of CDZ173 (0–20 µM) for the remaining 48 h. Subsequently, we added 10 ul/well CCK8 buffer to each well according to the instructions and at 37°C for 2 hours. The TriStar2 LB 942 multi-mode microplate reader was used to measure the absorbance at 450 nm (Bio-Tek Instruments, Winooski, VT, United States).

### Osteoclast formation assay *in vitro*

To observe whether CDZ173 affects the differentiation of osteoclasts, M-CSF-dependent BMMs were seeded at a density of 6×10^3^ cells per well in 96-well plates that contained full -MEM. To confirm that the cells adhered properly, they were placed there for an entire night. BMMs were simulated by employing a concentration of RANKL equal to 30 ng/ml in conjunction with an incremental doubling of CDZ173 (from 1.25, 2.5, and 5.0 μM, dose-dependent effect). The M-CSF, RANKL, and CDZ173-containing culture medium was changed every 2 days to promote the development of mature, multinucleated OCs. Detection of Tartrate-resistant acid phosphatase (TRAP) activity involved staining for the protein after cells were fixed in 4% paraformaldehyde for 15–20 min.

### Real-time PCR analysis

In order to explore the expression of particular OC genes at the conclusion of OC formation following the application of CDZ173, real-time PCR was utilized. The same reagents and settings for the real-time PCR apparatus were used as were described in the prior literature for the extraction of cell RNA (Li et al., 2020). Table 1 shows the related primer sets.

**TABLE 1:** The primer sets used are as follows.


TRACP	Forward:5′-TGTGGCCATCTTTATGCT-3'
	Reverse:5′-GTCATTTCTTTGGGGCTT-3′
DC-STAMP	Forward: 5′-TCT​GCT​GTA​TCG​GCT​CAT​CTC-3'
	Reverse: 5′-ACT​CCT​TGG​GTT​CCT​TGC​TT-3'
MMP-9	Forward: 5′-CGT​GTC​TGG​AGA​TTC​GAC​TTG​A-3'
	Reverse: 5′-TTG​GAA​ACT​CAC​ACG​CCA​GA-3′
CTSK	Forward: 5′-AGG​CGG​CTC​TAT​ATG​ACC​ACT​G-3'
	Reverse: 5′-TCT​TCA​GGG​CTT​TCT​CGT​TC-3'
C-fos	Forward: 5′-CCA​GTC​AAG​AGC​ATC​AGC​AA-3'
	Reverse: 5′-AAG​TAG​TGC​AGC​CCG​GAG​TA-3'
NFATc1	Forward: 5′-GGT​GCT​GTC​TGG​CCA​TAA​CT-3'
	Reverse: 5′-GAA​ACG​CTG​GTA​CTG​GCT​TC-3′
β-actin	Forward: 5ʹ-TCT​GCT​GGA​AGG​TGG​ACA​GT-3ʹ
	Reverse: 5′-CCT​CTA​TGC​CAA​CAC​AGT​GC-3ʹ

### Bone resorption pit assay

In the beginning, BMMs were plated at a density of 1×10^5^ cells per well into 6-well plates that contained a-MEM complete media. They were then subjected to the culture of an extra 30 ng/ml of RANKL until mature osteoclasts developed. Mature osteoclasts were isolated and then cultured for an additional 2 days in hydroxyapatite-coated 96-well plates (Corning, United States) containing varying doses of CDZ173. Lastly, the mature OCs were dissolved in 10% sodium hypochlorite for 15 min, followed by two washes in phosphate-buffered saline (PBS), and finally drying. Light microscopic images were captured for each well, and pit areas were quantified using ImageJ. BMM cells were implanted into 6-well plates containing α-MEM complete medium at a density of 1×10^5^ cells/well under 30 ng/ml RANKL and 30 ng/ml M-CSF culture until mature osteoclasts appeared on the third or fourth day.

### Podosome belt formation assay

To induce the development of fully functional, multinucleated OC, BMMs (8×10^3^ cells/well) were seeded into 96-well plates and grown in complete -MEM media with M-CSF (30 ng/ml), RANKL (30 ng/ml), and CDZ173 (1.25, 2.5, and 5 M). On day six, cells were washed, fixed with 4% paraformaldehyde, and permeabilized with 100 ml of 0.1% Triton X-100 in each well for 5 min. Nonspecific immunoreactivity was blocked with the 3% BSA in PBS. Podosome belts were stained by washing the cells and then incubating them with rhodamine-conjugated phalloidin for 1 h in a dark environment. Following this, the cells were washed with PBS a total of three times, and then counterstained with DAPI for a period of 5 minutes. After that, images were obtained through the employment of a fluorescent microscope (Leica, Germany).

### Osteoblastogenesis assay

We obtained bone mesenchymal stem cells (BMSc) from the long bone in 4–6-week-old male C57BL/6 mice. BMSc were grown in a humidified incubator at 37°C and 5% CO_2_ using a complete medium consisting of a-MEM supplemented with 10% (v/v) FBS, 1% (w/v) penicillin/streptomycin. To induce osteoblast differentiation, the grown BMSc (5×10^4^ cells/well) were seeded in 48-well plates and cultured in osteogenic medium (50 μg/ml ascorbic acid, 5 mM glycerol phosphate) with or without different concentrations of CDZ173. The culture medium was changed every other day, ALP staining was performed after 7 days of differentiation, and AR staining was performed after 21 days of differentiation.

### Western blotting

The molecular mechanism through which CDZ173 inhibits osteoclasts was analyzed by using western blotting. BMMs were plated at a density of 1.5 × 10^5^ cells/well in 6-well dishes. BMMs adhered overnight and were stimulated with RANKL for 0 (no stimulation), 5, 10, 20, 30, or 60 min after pretreatment with 5 µM CDZ173 or a vehicle control. The whole cells were then extracted in RIPA lysis buffer containing phosphatase inhibitor and phenylmethylsulfonyl fluoride (PMSF)for 30 min to obtain total proteins. Subsequently, an electronic imprinting device separated the proteins using SDS-PAGE gel and transferred them to a nitrocellulose (NC) membrane. The membrane was then sealed in 5% skimmed milk for 1 h and then incubated overnight at 4°C with specific primary antibodies: PI3K(1:1,000), phospho-PI3K(1:500), AKT (1:1,000), phospho-AKT (1:500), ERK (1:1,000), phospho-ERK (1:1,000) (1:500), JNK(1:1,000), phospho-JNK(1:500), p38 (1:1,000), phospho-p38 (1:500), NF-κB p65 (1:1,000), anti-phospho-NF-κB p65 (1:500), β-Actin (1:1,000), NFATc1 (1:100) and c-Fos(1:1,000). The next day, the membrane was washed three times with TBS containing 1% Tween for 5 minutes and then incubated with a fluorescent secondary antibody for 1 h. Finally, the membrane was washed three times with TBST, visualized with image quantla-4000 imaging system (GE Healthcare, Chicago, Illinois, United States), and the results were analyzed using the ImageJ software.

### Statistical analysis

Data was illustrated either as means ± standard deviation (SD), or the representative one with all independent triplicates. Statistical analysis among or within groups was conducted by one-way ANOVA tests using SPSS 19.0 software (SPSS Inc., United States).*p < 0.05, **p < 0.01, ***p < 0.001 was regarded as statistical significance.

## Results

### CDZ173 protected LPS-Induced calvarial osteolysis by inhibiting osteoclast activation *in vivo*

CDZ173 (Figure 1B), a selective PI3K inhibitor, has been used to treat immune diseases and arthritis (Hoegenauer et al., 2017). At the same time, it can alleviate blood-related tumors by inhibiting the PI3K-AKT signaling pathway (Hoegenauer et al., 2017; Rao et al., 2017) To explore the potential of CDZ173 (Figure 1B) to protect joint prosthesis loosening, we established a model of skull osteolysis induced by LPS to simulate bone loss caused by inflammation *in vivo*. Subsequently, we injected LPS and CDZ173 (2.5 mg/kg and 5 mg/kg) subcutaneously into the sagittal suture of the head in mice. Compared with the sham group, micro-CT showed an extensive bone loss in the calvaria of mice after LPS injection (Figure 1C). Compared with the LPS group, the mice treated with CDZ173 had reduced bone loss in the skull, and the recovery of bone volume/tissue volume (BV/TV) and trabecular bone separation (Tb. Sp) in the high dose (5.0 mg/kg) group was better than that in the low dose (2.5 mg/kg) group (Figures 1C–E). In terms of the bone morphological parameters, trabecular thickness (Tb. Th), there appeared to be a trend of recovery in the high-dose (5.0 mg/kg) group compared with the LPS group, although there was no significant difference due to the larger standard (Figure 1F).

**FIGURE 2:**
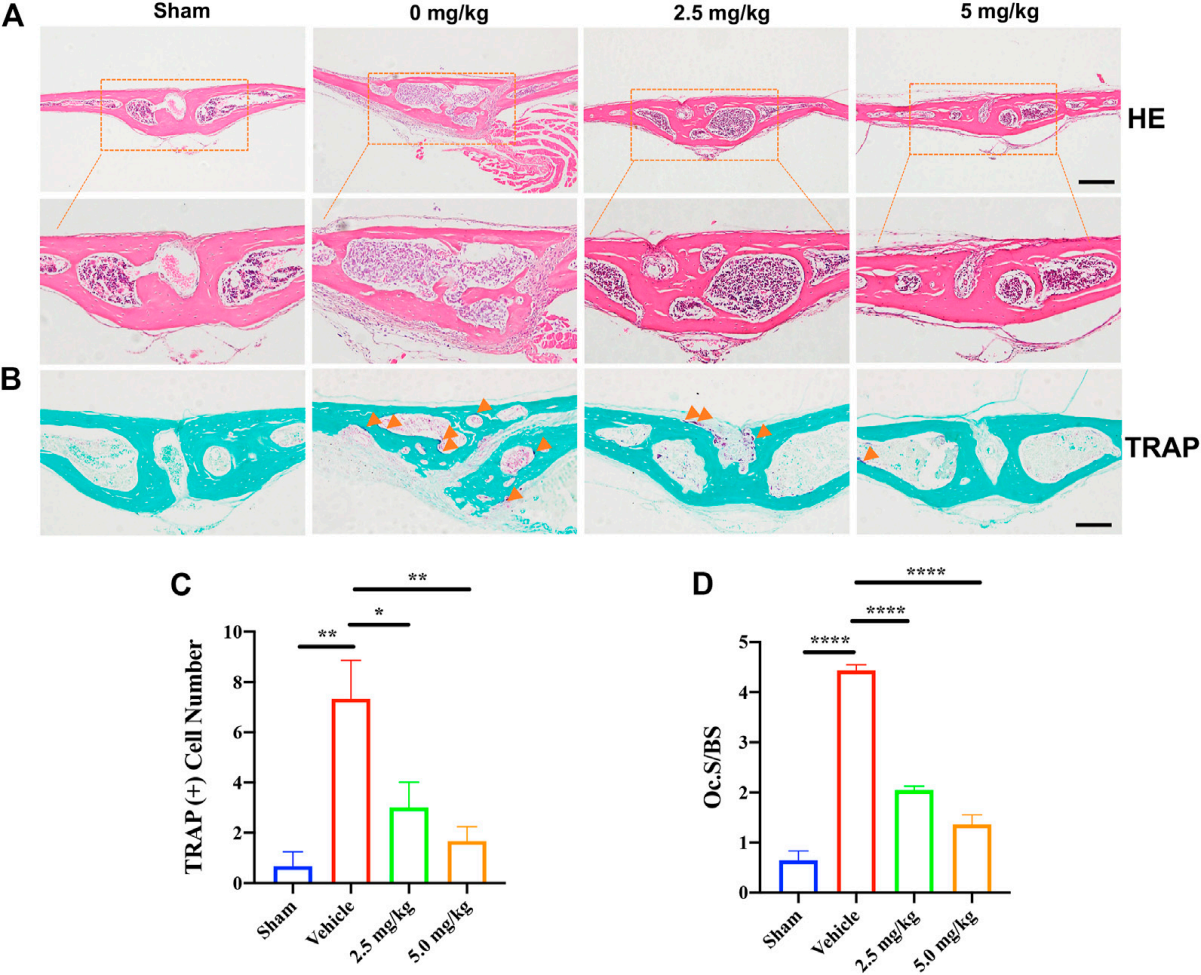
H&E and TRAP staining were performed on the calvarial tissue collected from the Sham, PBS vehicle, and CDZ173 treated groups. Representative images of calvaria stained with H&E**(A)** and TRAP**(B)**, histological assessment of **(C–D)**, and number of osteoclasts per field of tissue (TRAP^+^ Number) and (Oc.S/BS) in sections were shown (scale bar = 500 µM) (*p < 0.05, **p < 0.01, ****p < 0.0001).

Histological H&E staining further confirmed the protective effect of CDZ173 on LPS-induced bone erosion (Figure 2A). Additionally, histological TRAP staining and quantification showed that the number of osteoclasts on the bone surface was significantly reduced in the low-dose CDZ173 group (*p* = 0.0147) and the high-dose CDZ173 group (*p* = 0.0039) compared with the LPS group (Figures 2B,C), and the ratio of osteoclast surface to bone contact surface was also significantly reduced in the low-dose (*p* < 0.0001) and high-dose (*p* < 0.0001) CDZ173 groups (Figure 2D). Therefore, our data show that CDZ173 alleviates LPS-induced calvarial loss in mice by inhibiting the over-activation of osteoclasts *in vivo.*

### CDZ173 inhibited RANKL-induced osteoclast differentiation and had no toxic effect on BMMs *in vitro*

**FIGURE 3:**
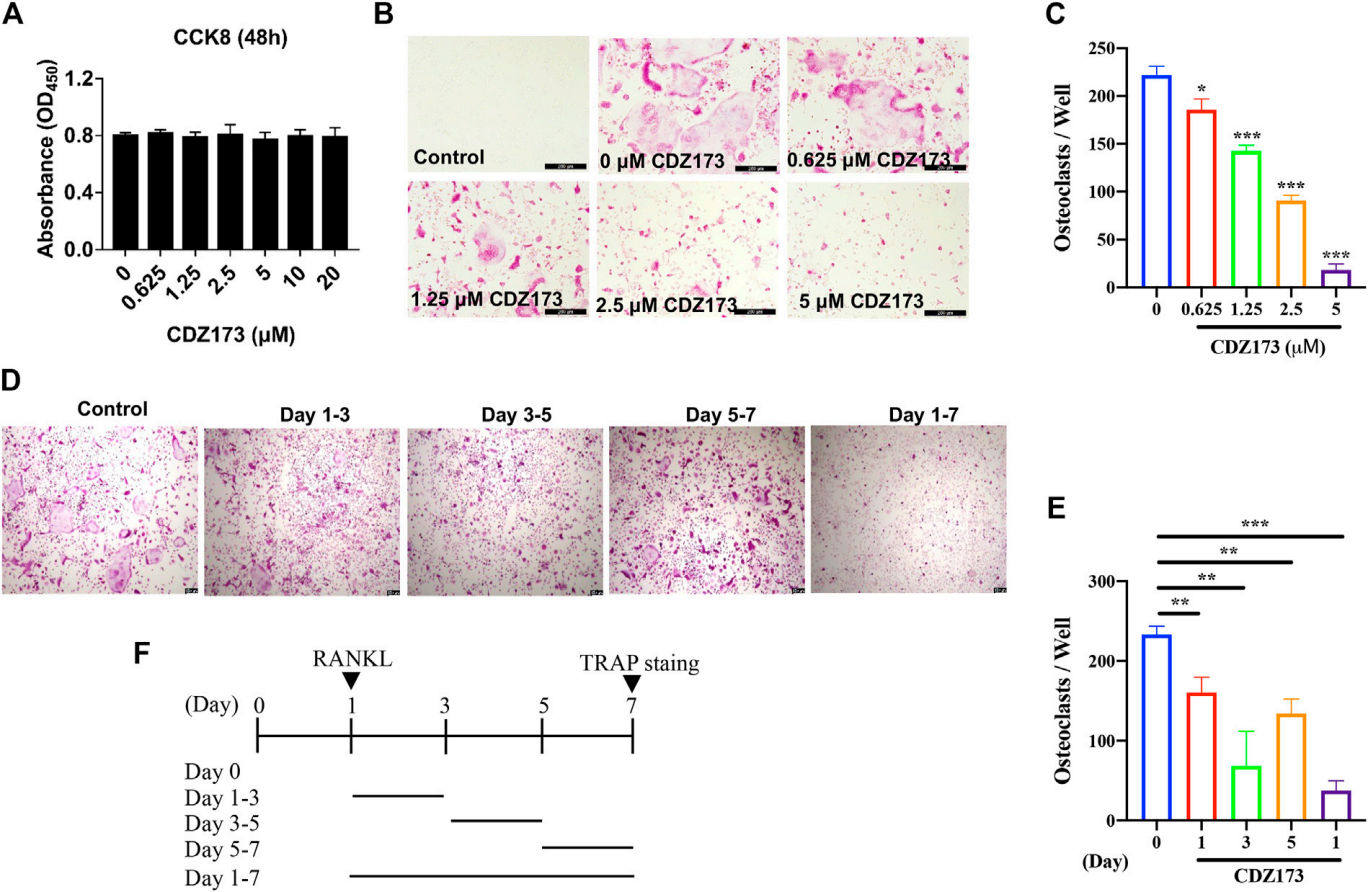
CDZ173 inhibited RANKL-induced OC formation without cytotoxic effects *in vitro*. **(A)** BMMs were treated with indicated concentrations of CDZ173 for 48 h and tested by CCK-8 assays. **(B)** BMMs were given the relevant doses of CDZ173, M-CSF (30 ng/ml), and RANKL (30 ng/ml) over the course of 5 days. After that, the cells were stained for TRAP after being fixed with 4% paraformaldehyde (scale bar = 200 µM). **(C)** The number of TRAP positive multinucleated OC (nuclei >3) were quantified (n = 3). **(D–F)** TRAP positive BMMs after the treatment with 5 µM CDZ173 for the indicated days during OC formation (scale bar = 100 µM). **(E)** The number of TRAP positive multinucleated OC (nuclei >3) were quantified (n = 3) (*p < 0.05, **p < 0.01, ***p < 0.001).

We previously determined the protective effect of CDZ173 on local calvarial osteolysis by inhibiting the activation of osteoclasts *in vivo*. Next, we explored the protective effect of CDZ173 on LPS-induced osteolysis *in vitro*. First, we evaluated the toxic effect of CDZ173 on BMMs using the CCK8 assay. As shown in Figure 3A, CDZ173 did not have any toxic effect on BMMs when the concentration of CDZ173 was below 10 μM. At the same time, we found that CDZ173 inhibited the number and size of osteoclasts under different concentrations (1.25, 2.5, and 5 μM) by RANKL-induced BMMs osteoclast differentiation in a dose-dependent manner, with the most significant inhibitory effect at 5μM, where osteoclast number decreased by about 90 percent (Figures 3B,C). To further explore at which stage CDZ173 inhibited osteoclasts, we set up the effect of CDZ173 on osteoclasts at different time gradients. We found that CDZ173 inhibited osteoclasts through TRAP staining and quantitative analysis. The best inhibitory effect is in the middle stage of cell differentiation (Figures 3D–F). Taken together, we identified that CDZ173 inhibited osteoclast differentiation by disrupting RANKL-induced metaphase signaling in BMMs.

### CDZ173 inhibited bone resorption and podosol actin belt formation

**FIGURE 4:**
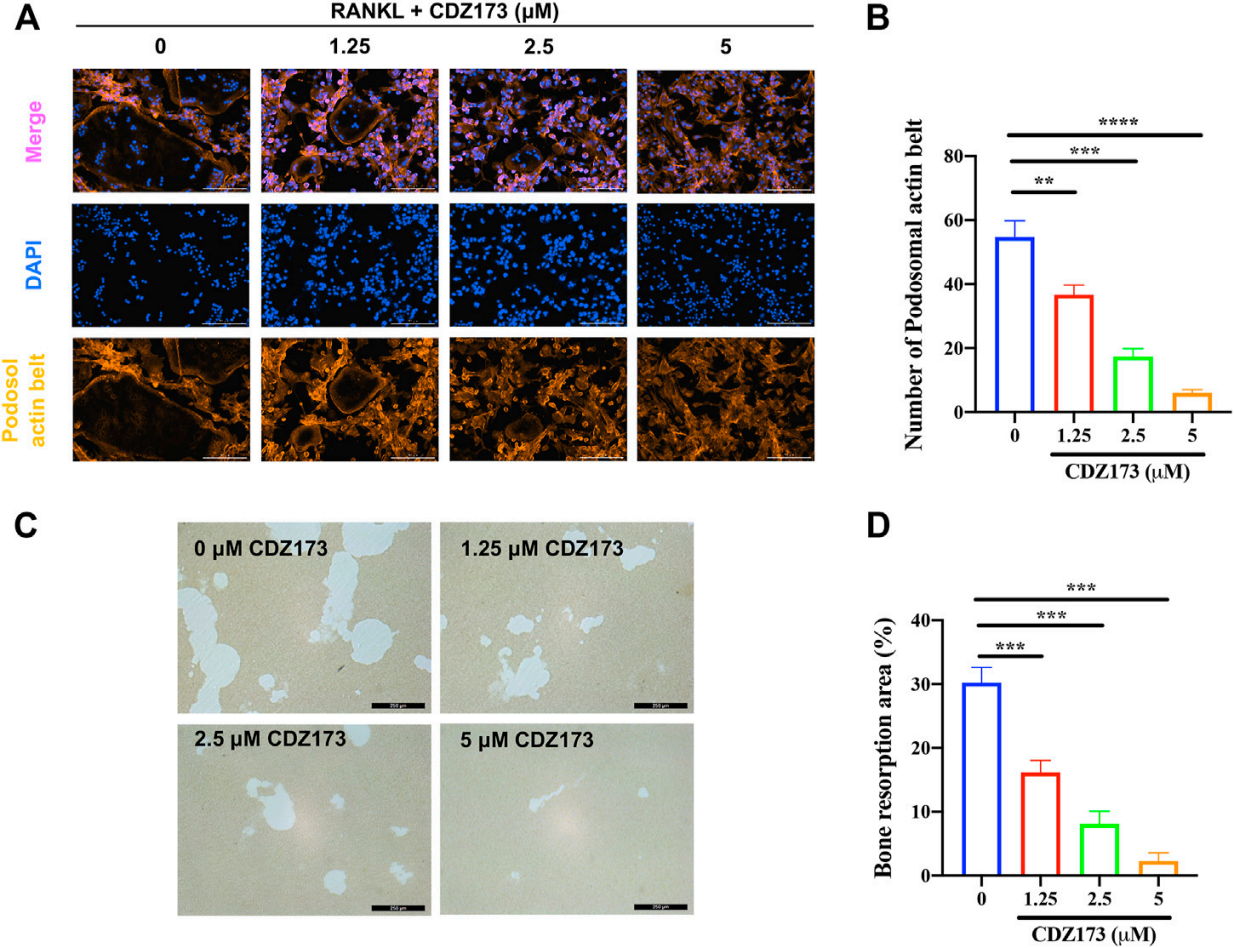
CDZ173 inhibited bone resorption and osteoclast fusion *in vitro* **(A)** BMMs were stimulated for 5 days with M-CSF (30 ng/ml) and RANKL (30 ng/ml) in the absence or presence of indicated concentrations of CDZ173, then fixed and immunostained for podosomeal actin belt. The representative image of the podosomal actin belt (Orange) in osteoclasts was captured by an immunofluorescence microscope. The nuclei (blue) were counterstained with DAPI (scale bar = 200 µM). **(B)** The number of podosomal actin belts in each group was quantified. **(C)** Mature OC were placed in hydroxyapatite-coated plates and treated for 24 h with 5 µM CDZ173. Attached cells were removed and micrographs of bone resorption pits were taken under a light microscope (scale bar = 250 µM). **(D)** The proportion of bone resorption area in total bone resorption area was quantified by ImageJ software (**p < 0.01, ***p < 0.001, ****p < 0.0001).

Both the development of the podosol actin belt and the resorption of bone tissue are necessary preconditions for the actualization of osteoclast function (Tan et al., 2017; Qiu et al., 2022). At the same time, in the above osteoclast differentiation TRAP staining experiment, we found that the osteoclasts generated following treatment with CDZ173 were noticeably smaller in comparison to those that had not been treated. As a result, we investigated this morphological shift further by labeling the podosol actin belt with immunofluorescence. The podosol actin belt is organized into a narrow actin band that surrounds one dormant nonpolarized osteoclast. This band serves to demarcate the boundaries of a single osteoclast. This feature can determine changes in osteoclast size, indicating impaired precursor cell fusion (Wang et al., 2019). Notably, as the concentration of CDZ173 increased, the number of podosol belts decreased in a concentration-dependent manner compared with the control group, especially at a CDZ173 concentration of (5 μM), the number of podosol actin belts decreased most significantly (Figures 4A,B). Next, we investigated the effect of CDZ173 on the bone resorption function of osteoclasts. As shown in Figures 4C,D, apatite absorption lacunae were reduced in size by CDZ173 in a concentration-dependent manner. Compared with the control group, the hydroxyapatite absorption lacunae after treatment with CDZ173 with a concentration of 5 μM decreased by nearly 30%. These findings imply that CDZ173 blocks the osteoclast function of RANKL-induced BMMs.

### CDZ173 inhibited osteoclast-specific gene expression

**FIGURE 5:**
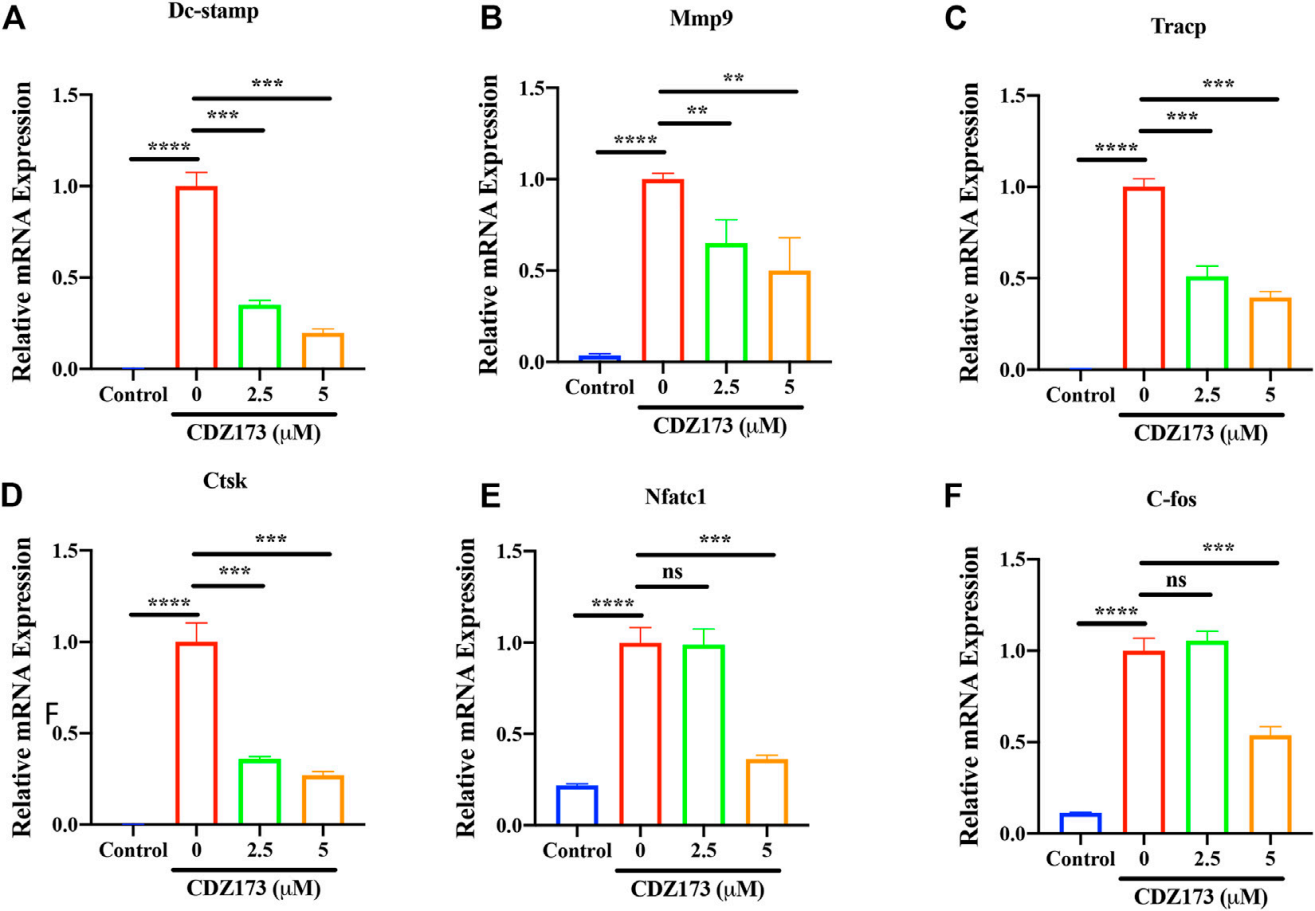
CDZ173 suppressed RANKL-induced expression of OC-Specific genes. BMMs were stimulated with M-CSF(30 ng/ml), and RANKL (30 ng/ml) in the absence or presence of indicated concentrations of CDZ173 for 5 days to form multinucleated TRAP positive OCs. Specific primers were used to perform real-time PCR for OC related genes **(A)** *Dc-stamp,* **(B)** *Mmp-9*, **(C)** *Tracp,* **(D)** *Ctsk,* **(E)** *Nfatc1* and **(F)** *C-fos* Gene expression levels of *Dc-stamp, Mmp9, Tracp, Ctsk, Nfatc1 and C-fos* were expressed relative to control group (**p < 0.01, ***p < 0.001, ****p < 0.0001).

Osteoclast-specific genes are up-regulated during RANKL-induced osteoclast formation (Zhou et al., 2015; Krieger et al., 2020). These genes include transcription factors c-Fos and NFATc1 in the osteoclast nucleus, dendritic cell-specific transmembrane protein (DC-STAMP), matrix metallopeptidase 9 (MMP 9), tartrate resistant acid phosphatase (Tracp), and cathepsin K (CTSK) involved in absorption function. Therefore, we detected the effect of CDZ173 on osteoclast-specific genes by quantitative fluorescence PCR. We identified that CDZ173 inhibited their expression in a dose-dependent manner (Figures 5A–F). Therefore, osteoclast development was suppressed and osteoclast-specific gene expression was decreased by CDZ173 *in vitro*.

### CDZ173 does not affect osteoblast differentiation and mineralization

**FIGURE 6:**
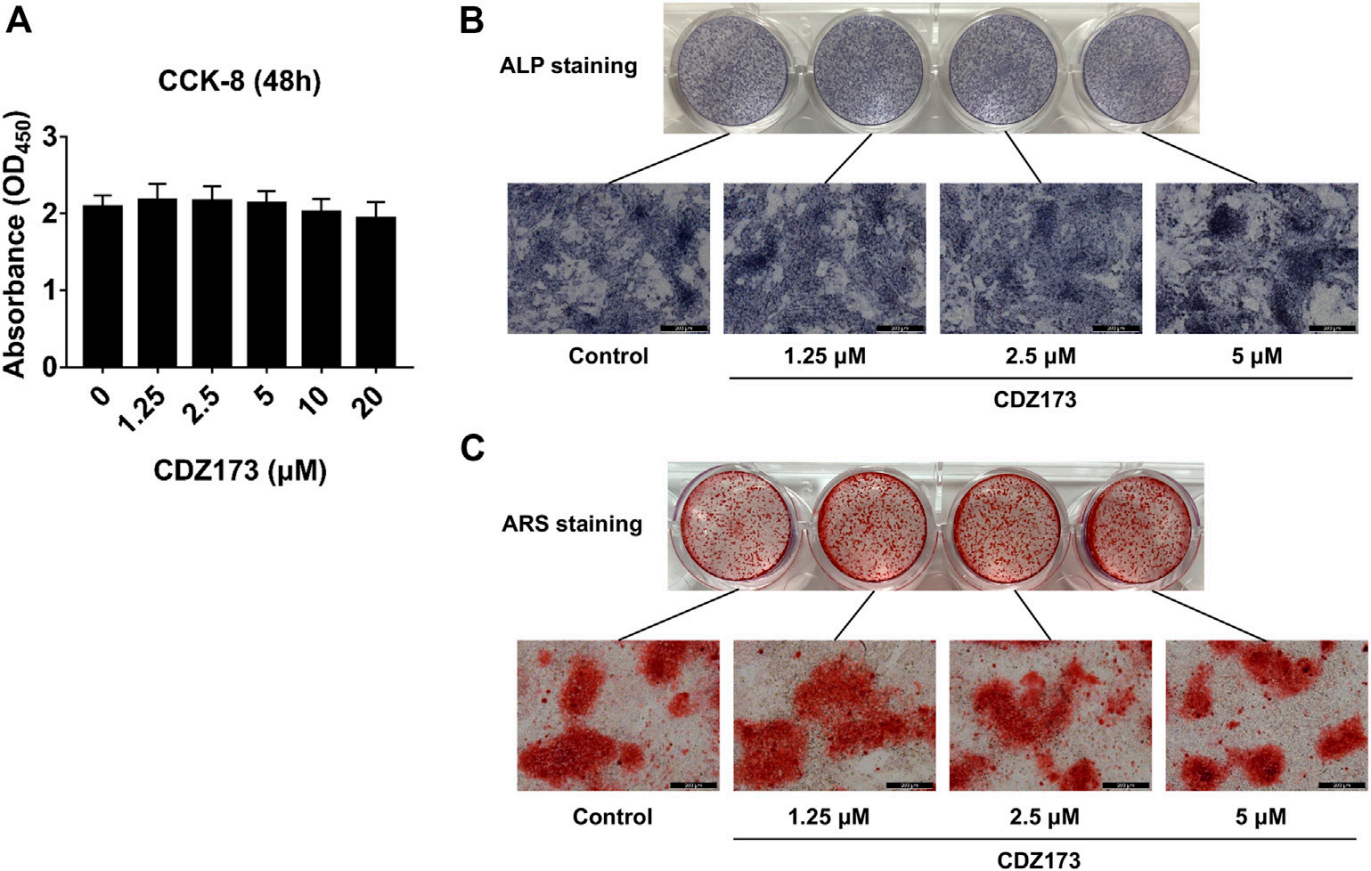
CDZ173 did not affect the differentiation and mineralization of osteoblasts. **(A)** For 48 h, BMSCs were exposed to the stated doses of CDZ173 before being evaluated using CCK-8 assays. **(B)** ALP staining in the absence or presence of CDZ173 (1.25, 2.5, and 5 μM) for 7 days (scale bar = 200 µM). **(C)** Alizarin Red S in the absence or presence of CDZ173 (1.25, 2.5, and 5 μM) for 21 days (scale bar = 200 µM).

We identified that CDZ173 protects LPS-induced local osteolysis by inhibiting the overactivation of osteoclasts. Therefore, we verified the effect of CDZ173 on osteoblasts by culturing osteoblasts *in vitro.* First, the toxic effects of CDZ173 (0, 10, 5, 2.5, and 1.25 μM) on BMSCs at different doses were determined using the CCK-8 reagent. The results showed that CDZ173 below 20 μM had no significant effect on the proliferation of BMSCs (Figure 6A). To explore the impact of CDZ173 on osteoblast formation, we carried out experiments of BMSC-induced osteoblast differentiation. BMSCs were cultured in an osteogenic induction medium, and different doses of CDZ173 (0, 1.25, 2.5, and 5 μM) were added for intervention. ALP and AR staining were performed on the cells after 7 and 21 days of culture, respectively (Figures 6B,C). The results demonstrated that CDZ173 had no appreciable impact on osteoblast differentiation and mineralization.

### CDZ173 inhibits osteoclast development by inhibiting PI3K-AKT and MAPKs signaling pathways

**FIGURE 7:**
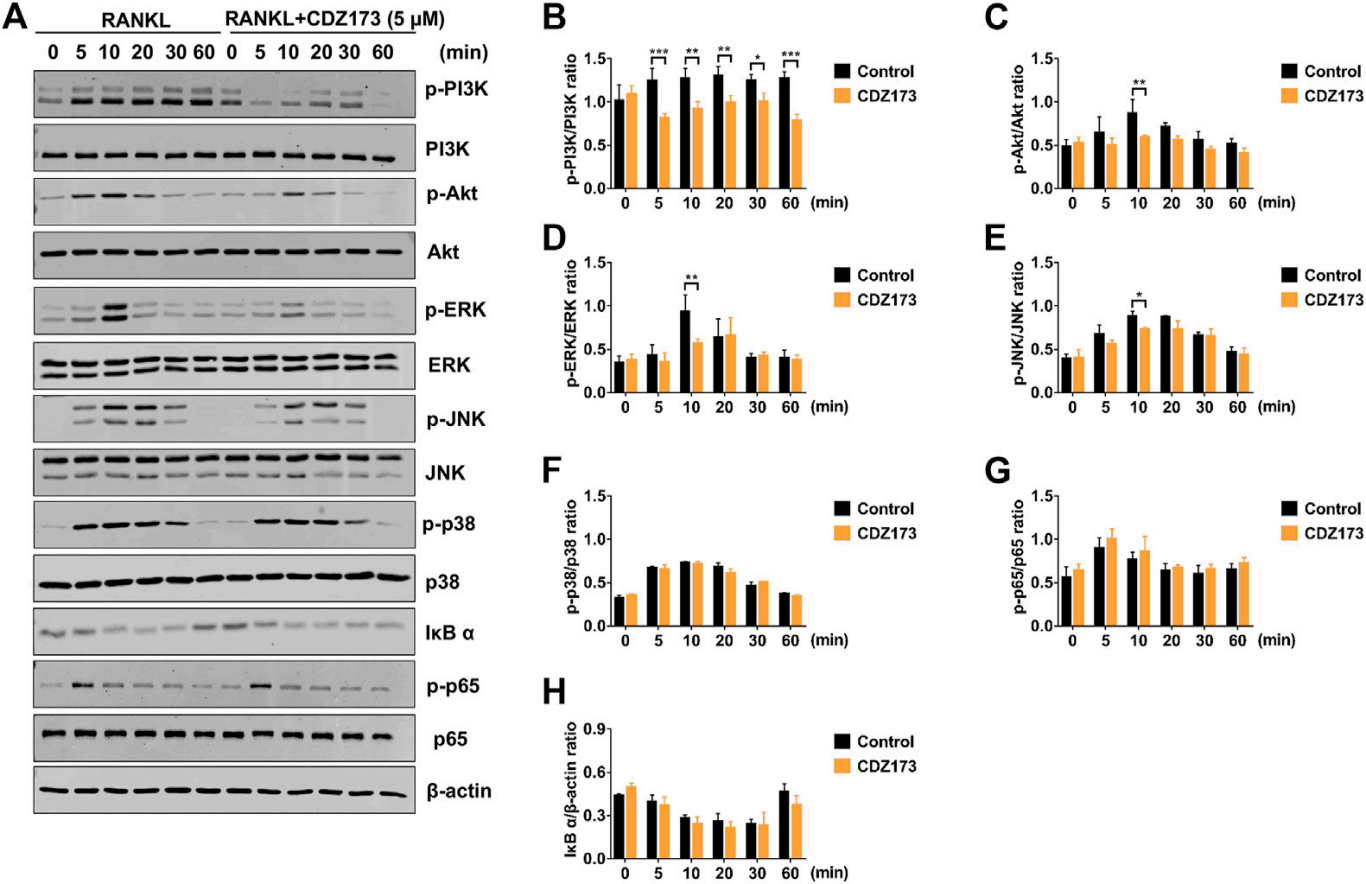
CDZ173 suppressed RANKL-induced PI3K-AKT and MAPKs but not NF-κB signaling pathways. **(A)** BMMs were pretreated for 4 h with or without 5 mM CDZ173, then stimulated for the given durations with RANKL (30 ng/ml). Cells were lysed, total proteins were extracted and subjected to western blot analysis using specific antibodies against p-PI3K, total PI3K p-AKT, total AKT, p-ERK, total ERK, p-JNK, total JNK, p-p38, total p38, p-p65, total p65 and IκBα. Antibody against β-actin was used as internal loading control. The relative densitometry of protein bands for **(B)** p-PI3K/total PI3K, **(C)** p-AKT/total AKT, **(D)** p-ERK/total ERK, **(E)** p-JNK/total JNK, **(F)** p-P38/total P38, **(G)** p-p65/total p65, and **(H)** IκBα/β-actin were quantified using ImageJ (n = 3). (*p < 0.05, **p < 0.01, ***p < 0.001).

To further clarify the potential molecular mechanism of CDZ173 involved in osteoclast differentiation and function, we first detected the PI3K-AKT classical signaling pathway of RANKL-induced osteoclast formation. After BMMs were stimulated by RANKL, western blot analysis demonstrated that the phosphorylation levels of PI3K and AKT increased significantly, reaching the peak at 5 and 10 min, respectively. However, after treatment with CDZ173, the phosphorylation levels of PI3K and AKT decreased significantly, while there was no significant change in total protein (Figures 7A,C). Previous research has demonstrated a critical function for the three main MAPK subfamilies (JNK, ERK, and p38) in osteoclast activity and development (Fang et al., 2020). At the same time, our western blot results showed that the phosphorylation of ERK, JNK, and p38 increased significantly under the stimulation of RANKL, and all three peaked in 10 min. However, after treatment with CDZ173, the phosphorylation of ERK and JNK decreased but had no impact on the phosphorylation of p38 (Figures 7A,D–F). We also observed the effect of CDZ173 on NF-κB. CDZ173 did not affect B cell inhibitory factor α (IκBα) degradation or p65 phosphorylation (Figures 7A,G,H), suggesting that CDZ173 does not affect NF-κB in the early signaling of the osteoclast process. These results demonstrated that CDZ173 attenuates osteoclast formation by inhibiting the binding of PI3K-AKT and MAPK signals.

### CDZ173 inhibits the transcription factors NFATc1 and c-fos in the osteoclast nucleus to weaken osteoclast differentiation

**FIGURE 8:**
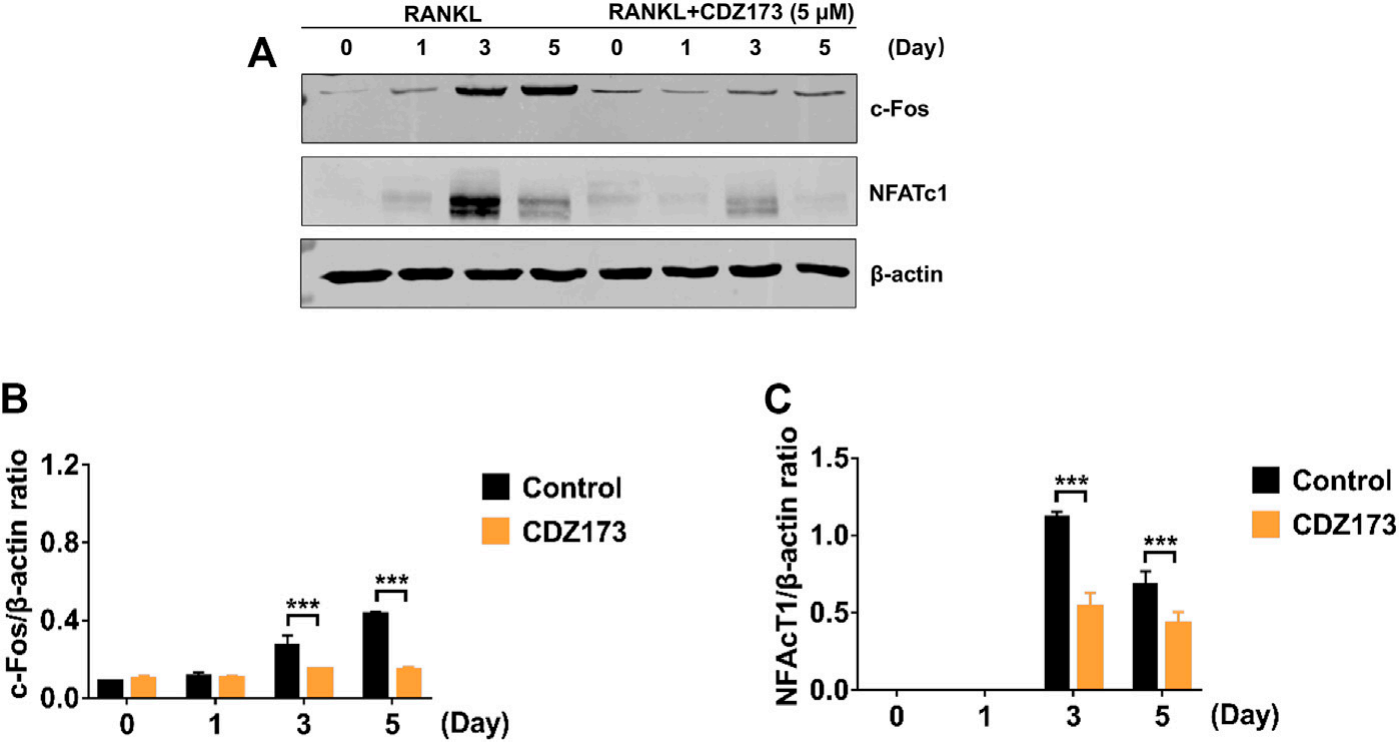
CDZ173 inhibited RANKL induced C-fos and NFATc1 transcriptional activity. **(A)** BMMs cultured with M-CSF(30 ng/ml) and RANKL (30 ng/ml) absence or presence of 5 μM CDZ173 for 1, 3 or 5 days were lysed and total cellular protein extracts were subjected to western blot analysis using specific antibodies to NFATc1, c-Fos and β-actin. The relative densitometry of protein bands for **(B)** c-Fos and **(C)** NFATc1 against β-actin were quantified using ImageJ (n = 3) (***p < 0.001).

**FIGURE 9:**
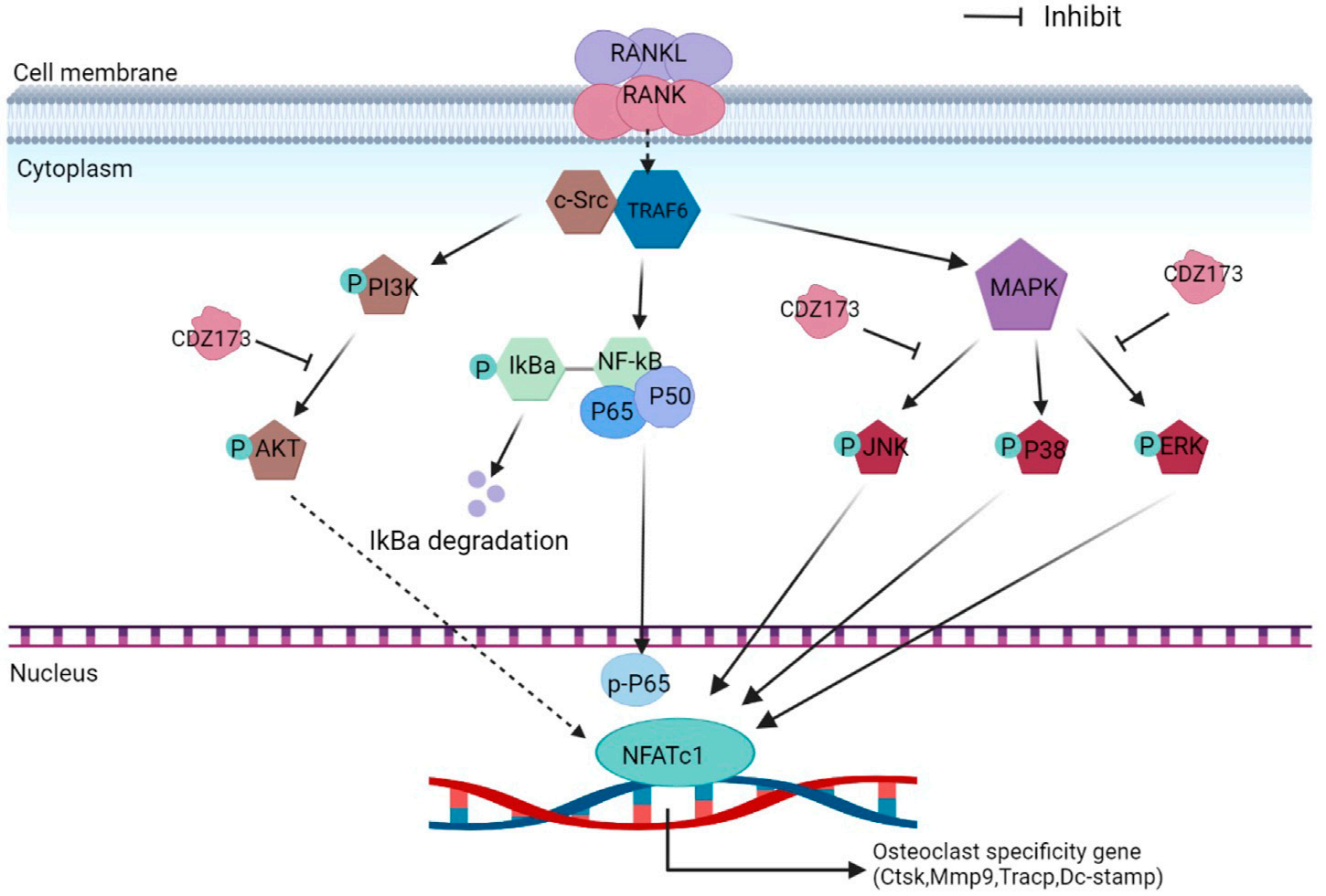
Schematic diagram of the suppressing influence of CDZ173 on the formation of osteoclasts.

During osteoclast development, activation of the PI3K-AKT, NF-κB, and MAPK signaling cascades are critical for efficient activation of downstream nuclear transcription factors such as c-Fos and NFATc1, which is a direct downstream transcription factor of c-Fos target (Lin et al., 2020; Kim et al., 2021). As a crucial distal transcription factor, NFATc1 regulates the transcription of many osteoclast marker genes and promotes the formation, fusion, and resorption of osteoclasts. Our earlier results determined that CDZ173 affects the osteoclasts early signaling pathways MAPK and PI3K-AKT. Therefore, the role of CDZ173 in the late development of osteoclasts was observed with these added investigations. Our findings revealed that the expression of transcription factor NFATc1 in the nucleus of osteoclasts peaked on the third day, and the expression of c-Fos peaked on the fifth day (Figure 8A). In contrast, the expression of NFATc1 and c-Fos were significantly down-regulated in the group treated with CDZ173 (Figures 8B,C). The above results indicated that the blockade of early PI3K-AKT, ERK, and JNK signaling pathways by CDZ173 effectively inhibited the induction and transcriptional activities of c-Fos and NFATc1 proteins, thereby contributing to the anti-osteoclast effect of CDZ173 small molecules (Figure 9).

## Discussion

Osteoclasts are responsible for resorbing bone matrix, in which osteoblasts differentiate into osteocytes (Udagawa et al., 2021). Thus, the mutually beneficial interaction of these 2 cells ensures that the structural integrity of skeletal tissue is kept in the best possible condition (Mediero and Cronstein, 2013). However, when osteoclasts are overactivated and gradually become dominant, bone resorption is more substantial than bone formation, leading to osteolytic diseases, including rheumatoid arthritis, periodontitis, aseptic sterility after joint replacement, and bone loss induced by prosthesis loosening (Zhu et al., 2021; Gao et al., 2022). Notably, joint replacement is still the most direct and effective surgery for patients with advanced bone disease. However, aseptic prosthesis loosening brought on by inflammatory osteolysis carried by postoperative periprosthetic infection is one of the main causes of joint replacement failure. It is still a difficulty faced by clinicians that also brings pain to patients and increases their economic burden (Liu et al., 2022). Bacterial endotoxin, which contaminates prostheses, has been identified as a significant factor in osteolysis caused by debris, as it enhances wear particle reactivity and its pro-inflammatory effects (Kandahari et al., 2016). Studies have shown that prosthetic loosening wear particles and bacterial product-related inflammatory responses activate the immune system to secrete pro-osteoclastogenic cytokines (Kandahari et al., 2016). This process enhances osteoclast recruitment and activity, ultimately promoting the dominance of bone resorption in bone metabolism, resulting in bone destruction in the periprosthetic area (Kandahari et al., 2016; Yan et al., 2018). Therefore, modulating abnormal osteoclast activity is an effective strategy to prevent inflammatory bone loss.

Added compounds have been studied or developed for treating osteolytic diseases, including calcitonin, cathepsin-K inhibitors, bisphosphonates, anti-RANKL antibodies, and some Chinese herbal medicines (Miller, 2009; Kuritani et al., 2018; Wu et al., 2019). However, in some cases, there is still a risk of severe side effects, including the presence of cardiovascular events and nephrotoxicity. As a potent PI3K-selective small molecule inhibitor with suitable properties and efficacy, CDZ173 can be used as a therapeutic agent for immune-mediated diseases and is currently in clinical trials for APDS and primary Sjögren’s syndrome (Rao et al., 2017; Jamee et al., 2020). In addition, oral CDZ173 has effectively decreased lymphocyte proliferation in clinical experimental studies (Rao et al., 2017). Furthermore, CDZ173 effectively inhibited the production of antigen-specific antibodies and alleviated disease symptoms in a rat collagen-induced arthritis model (Hoegenauer et al., 2017). These results indicate that CDZ173 has excellent anti-inflammatory properties, although it is unknown whether CDZ173 participates in inflammatory osteolysis. In the present study, we employed LPS to induce local osteolysis in the mice calvaria to mimic inflammation-induced bone loss disease. Micro-CT results showed that compared with the vehicle group, the BV/TV in the CDZ173-treated drug group was significantly increased, and the porosity also decreased, indicating a significant improvement in CDZ173 bone loss. At the same time, the TRAP staining of histological sections showed that the osteoclasts in the drug group were significantly reduced after CDZ173 treatment compared with the vehicle group. Therefore, we demonstrate that the protective effect of CDZ173 against LPS-induced calvarial bone loss is by inhibiting osteoclast activation *in vivo*.

Osteoclasts play a central role in forming and regulating bone mass and are the main resorbing cells of bone (Boyce, 2013; Meng et al., 2022). The initial process of bone resorption is the attachment of osteoclasts to the bone matrix, resulting in polarization followed by a fold of the cytoplasm and membrane into the ruffled edge, which attaches to the bone surface and secretes H+ through the proton pump on the ruffled edge (Takayanagi, 2021). The extracellular environment becomes acidified from these steps, thereby degrading the minerals of bone tissue, and its secreted cathepsin K degrades the bone matrix (Roodman, 1991; Takayanagi, 2021). Our *in vitro* osteoclast differentiation experiments showed that CDZ173 inhibited osteoclast differentiation in a concentration-dependent manner, and CDZ173 significantly inhibited the expression of osteoclast-specific genes. Then, we investigated the effect of CDZ173 on the resorption function of osteoclasts using bone-mimicking hydroxyapatite cell plates. The results showed that the bone resorption area of hydroxyapatite plates treated with CDZ173 was significantly reduced, suggesting that CDZ173 could inhibit the function of osteoclasts that lead to absorbing bone matrix. Meanwhile, we performed immunofluorescence staining experiments on the osteoclast podosol actin belt, and the results found that the size of the podosol actin belt was significantly reduced after CDZ173 treatment. At the same time, our ALP and AR staining conclusively confirmed that CDZ173 does not affect osteoblasts *in vitro*.

RANKL is extremely important during osteoclastogenesis. Its combination with cognate receptor RANK recruits the adaptor protein TRAF6 to rapidly trigger a series of downstream signaling events, mainly NF-κB, MAPK, and PI3K-AKT, that drive osteoclast precursor cells to differentiate and fuse into multinucleated giant cells and subsequently exert bone resorption (An et al., 2019; Xin et al., 2020). Results have shown that PI3K-AKT signaling is essential in osteoclast differentiation and survival (Xin et al., 2020; Zhao et al., 2020). Briefly, the RANKL-RANK interaction can activate PI3K signaling molecules, which activate AKT to promote its phosphorylation to p-AKT, thereby activating the PI3K-AKT signaling pathway to enhance the expression of NFATc1 and nuclear export (Xin et al., 2020). Collectively, these steps promote the inhibition of osteoclast differentiation (Xin et al., 2020). A previous study showed that the inhibition of PI3K-AKT by the PI3K inhibitor LY294002 reduced osteoclast formation and attenuated the expression of the NFATc1 transcription factor (Zhao et al., 2020), suggesting that the PI3K-AKT-NFATc1 signaling axis is essential for RANKL-induced osteoclastogenesis. CDZ173 is a selective small-molecule PI3K inhibitor (Hoegenauer et al., 2017). To study how CDZ173 regulates osteoclast formation, we performed western blot experiments to analyze its effects on PI3K-AKT, NF-κB, and MAPK signaling pathways. First, we applied western blotting to detect whether CDZ173 affected the phosphorylation status of PI3K and AKT. The results in western blot analysis indicated that CDZ173 treatment significantly improved RANKL-induced activation of the PI3K-AKT pathway.

When RANKL is stimulated, the classic signaling pathways in osteoclasts that respond include NF-κB and MAPK(An et al., 2019; Guo et al., 2020). However, our protein band analysis results indicated that CDZ173 responded to RANKL stimulation but did not inhibit the activation of NF-κB. Treatment of cells with CDZ173 did not affect IκBα degradation, p65 phosphorylation, and NF-κB transcriptional activity. This indicated that the effect of CDZ173 on the formation of RANKL-stimulated osteoclasts was not achieved by acting on the NF-κB signaling pathway. Activation of three MAPK signaling pathways, JNK, p38, and ERK, is important in efficiently stimulating osteoclastogenesis. Notably, the ability of monocyte precursors produced from JNK1 or ERK1 mutant mice to develop into the osteoclast lineage is diminished (He et al., 2011; Sadangi et al., 2020). Furthermore, ERK is essential for osteoclast survival and cell polarity maintenance during bone resorption. JNK signaling is essential for controlling osteoclast development, differentiation, and apoptosis. In contrast, p38 signaling is mainly involved in osteoclast production rather than activity. In the current investigation, we discovered that CDZ173 strongly decreased the activation of ERK and JNK signaling, but had no discernible impact on the activation of p38. Additionally, CDZ173 dramatically reduced the expression of c-Fos and NFATc1 according to our western blotting results, and our RT-PCR results similarly demonstrated that CDZ173 administration greatly reduced c-Fos and NFATc1 mRNA expression. The above results suggest that CDZ173 affects the formation of osteoclasts by inhibiting the activation of transcription factors c-Fos and NFATc1.

Collectively, we determined that CDZ173 attenuated LPS-induced calvarial bone loss and demonstrated this protective effect *in vitro* and *in vivo* due to CDZ173 inhibiting osteoclast activation without affecting osteoblast activation. Meanwhile, we demonstrated that CDZ173 inhibited osteoclast differentiation by downregulating the expression of Dc-stamp, Trap/Acp5, Mmp-9, and Ctsk genes by inhibiting RANKL-activated PI3K-AKT, ERK, and JNK signaling pathways *in vitro*. Combining the pharmacokinetics and safety evaluation of CDZ173 in clinical concept studies and the potential of CDZ173 for the treatment of immune diseases, we believe that CDZ173 is a promising drug for the treatment of osteoclast-mediated bone loss disease in the clinic.

## Data Availability

The original contributions presented in the study are included in the article/Supplementary Material, further inquiries can be directed to the corresponding authors.

## Abbreviations

ALP, Alkaline phosphatase; AR, Alizarin red; BMMs, Bone marrow macrophages; BMSc, Bone mesenchymal stem cells; BV/TV, Bone volume/tissue volume; CCK8, Cell counting kits 8; CTSK, Cathepsin K; DC-STAMP, Dendritic cell-specific transmembrane protein; H&E, Hematoxylin-Eosin; IκBα, B cell inhibitory factor α; LPS, Lipopolysaccharide; M-CSF, Macrophage colony-stimulating factor; MMP9, Matrix metallopeptidase 9; μCT, Microcomputed tomography; NC, Nitrocellulose; NFATc1, Nuclear factor of activated T-cell cytoplasmic 1; OC, Osteoclast; PMSF, Phosphatase inhibitor and phenylmethylsulfonyl fluoride; RANKL, Receptor activator of nuclear factor-κB ligand; RANK, Receptor activator of nuclear factor-κB; Tb. Sp, Trabecular bone separation; Tb.Th, Trabecular thickness; TLR4, Toll-like receptor 4; TRAF6, Tumor necrosis factor receptor-associated factor 6; TRAP, Tartrate-resistant acid phosphatase.
